# Metal Coordination‐Mediated Functional Grading and Self‐Healing in Mussel Byssus Cuticle

**DOI:** 10.1002/advs.201902043

**Published:** 2019-10-24

**Authors:** Quan Xu, Meng Xu, Chun‐Yu Lin, Qiang Zhao, Rui Zhang, Xiaoxiao Dong, Yida Zhang, Shouceng Tian, Yu Tian, Zhenhai Xia

**Affiliations:** ^1^ State Key Laboratory of Heavy Oil Processing Beijing Key Laboratory of Biogas Upgrading Utilization Harvard SEAS‐CUPB Joint Laboratory on Petroleum Science College of New Energy and Materials Science China University of Petroleum‐Beijing Beijing 102249 China; ^2^ Department of Orthopedics General Hospital of Chinese People's Liberation Army Beijing 100853 China; ^3^ Department of Materials Science and Engineering University of North Texas Denton TX 76203 USA; ^4^ School of Chemistry and Chemical Engineering Huazhong University of Science&Technology Wuhan 435000 China; ^5^ State Key Laboratory of Tribology Tsinghua University Beijing 100084 China

**Keywords:** density functional theory, iron complex, mussels, self‐healing, tensile tests

## Abstract

Metal‐containing polymer networks are ubiquitous in biological systems, and their unique structures enable a variety of fascinating biological behaviors. Cuticle of mussel byssal threads, containing Fe‐catecholate complexes, shows remarkably high hardness, high extensibility, and self‐healing capability. Understanding strengthening and self‐healing mechanisms is essential for elucidating animal behaviors and rationally designing mussel‐inspired materials. Here, direct evidence of Fe^3+^ and Fe^2+^ gradient distribution across the cuticle thickness is demonstrated, which shows more Fe^2+^ inside the inner cuticle, to support the hypothesis that the cuticle is a functionally graded material with high stiffness, extensibility, and self‐healing capacity. The mechanical tests of the mussel threads show that both strength and extensibility of the threads decrease with increasing oxygen contents, but this property degradation can be restored upon removing the oxygen. The first‐principles calculations explain the change in iron coordination, which plays a key role in strengthening, degradation, and self‐healing of the polymer networks. The oxygen absorbs on metal ions, weakening the iron‐catecholate bonds in the cuticle and collagen core, but this process can be reversed by sea water. These findings can have important implications in the design of next‐generation bioinspired robust, highly extensible materials, and catalysis.

## Graphical abstract

Evidence of Fe^3+^ and Fe^2+^ gradient distribution across the cuticle thickness of bypass thread is provided, which supports that the cuticle is a functionally graded material with high stiffness, extensibility, and self‐healing capacity. The oxygen absorbed on metal ions can weaken the iron‐catecholate bonds in the cuticle and collagen core, but cuticle can heal itself in sea water.

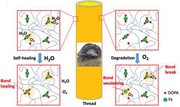

Nature‐evolved organisms have produced a variety of unique materials that possess extraordinary abilities or characteristics, such as high strength and toughness, self‐healing, efficient energy conversion, brilliant structural colors, intelligence, and so on, which provides inspiration for researchers to create a variety of advanced materials with similar properties.^1^ The byssus of marine mussels is one of the remarkable examples and has attracted considerable attention for their unique iron‐clad fiber structures, high stability, outstanding extensibility, and self‐healing properties.^2^ It is well known that mussels can adhere to almost all the surfaces including polytetrafluoroethylene, one of the inert surfaces, using their adhesive byssus.^3^ To overcome the risk of dislodgement by incoming waves, the byssus must be strong, tough, resistant to bacteria, and stable over extreme environments.^4^, ^5^ Over the time, mussels have evolved adoptive byssal threads with exceptional toughness, self‐healing capabilities, high elastic modulus (≈100 MPa), high hardness (≈100 MPa for *Mytilus galloprovincialis*), and high extensibility (≈100%).^6^, ^7^

The extraordinary properties of the byssal threads stem from their unique hierarchical structures that combine collagenous cores surrounded by protective cuticle, mainly containing catecholic amino acid 3,4‐dihydroxyphenylalanine (DOPA) and iron, which form crosslinking iron‐catechol structures. Here, the iron is essential to the mechanical and self‐healing properties. It was reported that the cuticle showed twofold decrease in hardness after removing metals from it.^8^ Metal‐coordination in the crosslinks also plays a unique role in forming different structures (bis‐complex coordination, Fe^2+^, for a linear crosslinking, and tris‐complex coordination, Fe^3+^, for 3D crosslinking), response to the environment (e.g., pH), and functioning as sacrificial bonds to prevent catastrophic material failure.^9^ However, the detailed enhancing and self‐healing mechanisms of the iron are still unclear due to the lack of insights into the reactions at the molecular level. On the other hand, although approaches for making metal coordination with enhanced mechanical and self‐healing properties have been developed,^10^ most strategies are limited to soft systems such as hydrogels (*E* < 50 MPa) while the cuticle shows hardness up to 1 GPa and stiffness up to 2 GPa.^3^, ^11^ To design a promising artificial surface coatings with high stiffness, extensibility, and self‐healing capability, it is important to understand the molecular strengthening and self‐healing mechanisms in mussel cuticles.

In this contribution, we have demonstrated the first direct evidence of Fe^2+^ existing inside the cuticle, suggesting that the cuticle is a functionally graded material. The transition between Fe^2+^ and Fe^3+^ plays a key role in maintaining high stiffness, extensibility, and self‐healing of the cuticle. Our experiment combined with the first‐principles and finite element simulations demonstrated that oxygen might weaken and even break the Fe‐DOPA bonds, which led to the degradation of mussel threads, but the weakened and broken bonds in Fe‐DOPA complexes could be restored spontaneously in the presence of sea water. These findings provide a theoretical base of designing next‐generation mussel‐inspired coating surfaces which have high stiffness, extensibility, and self‐healing capacity.

The macro/microstructures of mussel threads and cuticle were examined using advanced microscopes. **Figure** **1**A shows a mussel and its threads/byssus adhered to a wood surface. The threads have three regions, core, cuticle, and plaque,^12^ formed in the groove of the mussel foot by injection molding of precursors secreted from cells around the groove line and the whole casting process takes only up to 3 min (Figure 1B). Figure S1 in the Supporting Information captured the moment of a mussel forming a fresh byssus. When viewing the cross‐section of the threads under the scanning electron microscope (SEM), a thin protective coating (cuticle) with a thickness around 1–5 µm was observed, with a function of protecting inside fibers (Figure 1C,D). Transmission electron microscope (TEM) reveals that *M. galloprovincials* cuticle consists of biphasic granules in homogenous matrix. The granules have a size ranging between 0.5 and 2 µm and comprise around 50% of the cuticle volume (Figure 1E,F). The cuticle can resist up to 80% of a strain along the thread length^7^ and keep its shape under extreme environment (Figure S2, Supporting Information). The edge of the cuticle was further confirmed under confocal laser microscope from Figure S3 in the Supporting Information with wavelengths at 405, 488, and 633 nm, respectively. A clear edge band was observed at the edges of the thread.^13^ Interestingly, whereas the emission fluorescence is at 405 nm, we also observed strong fluorescence with wavelength at 633 nm (Figure S3, Supporting Information).

**Figure 1:**
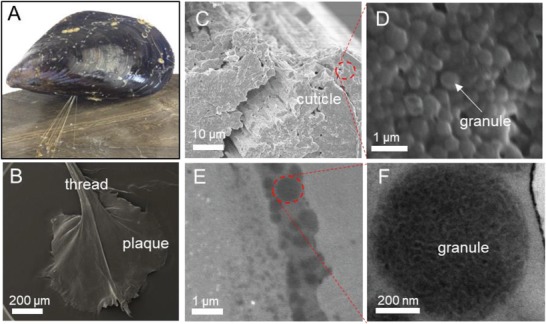
Structures of mussel byssus thread at different length scales. A) a mussel attached by a byssus to wood and B) SEM of the mussel gives the relationship of mussel thread, cuticle, and plaque. C) SEM of *M. galloprovincialis* distal thread with delamination of cuticle coating. D) Enlarged SEM image of granule on cuticle with size at around 500 nm. E) TEM of transverse cross‐section of *M. galloprovincialis* distal thread cuticle. F) High‐resolution TEM image of granule illustrating composite structure as well as interior granular structure.

We further examined the molecular structures and coordination states of iron in the cuticle. Mussel cuticle is mainly made up of repetitive sequences π‐rich proteins,^14^ dominated by iron‐DOPA complexes and collectively termed mussel foot protein (mfp‐1) (Figure S4, Supporting Information).^13^ To examine the oxidation states of iron ions in cuticle, a fresh thread was collected and analyzed by C K‐edge X‐ray absorption near edge spectroscopy (XANES) and X‐ray photoelectron spectroscopy (XPS). The depth profiles of Fe^3+^ and Fe^2+^ elements were conducted at the surface, 20, 40, and 60 nm in depth of the cuticle (**Figure** **2**A) and the proportional distributions of Fe^3+^ and Fe^2+^ elements were measured (Figure 2C and Figure S5, Supporting Information), the etching depth was confirmed by atomic force microscope (AFM; Figure S6, Supporting Information). It was found that the Fe peak (≈709 eV) for 40 nm depth was shifted to lower energy compared with the surface profile. This implies that Fe ions in low oxidation state, such as Fe^2+^, exist in the inner layer of mussel threads, while those in high oxidation state, such as Fe^3+^, tend to be enriched on the thread surface. This phenomenon was further confirmed by the XPS experiment, as shown in Figure 2B. The XPS of Fe 2P_1/2_ and Fe 2P_3/2_ clearly proved the transformation between Fe^3+^ and Fe^2+^ from the surface to 40 nm depth. Furthermore, our experimental results show that Fe^3+^ ions are enriched on the thread surfaces, especially on the old thread, which implies that this Fe oxidation process continues during the lifetime of each thread. These evidences strongly support the hypothesis that the cuticle is a functionally graded material, and this feature may strongly enhance the properties of the threads.

**Figure 2:**
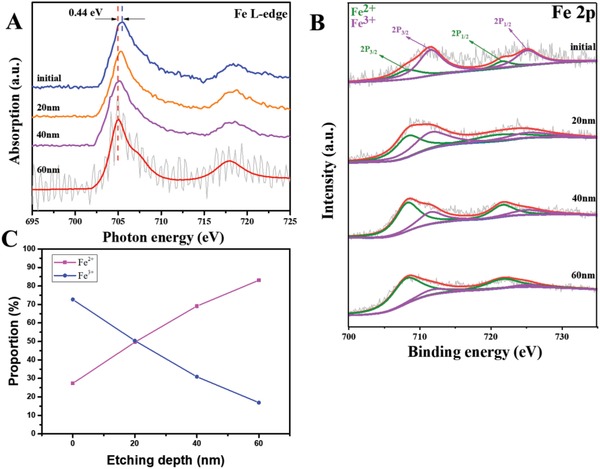
Oxidation state of iron in the cuticle of a fresh thread, analyzed by XANES and XPS. A) XANES spectra mapping of the surface and 20, 40, 60 nm etching cuticle on mussel thread, and B) XPS analysis of surface and 20, 40, 60 nm etching thread. The shift of the peaks at different depths implies that a lower oxidation state of Fe, such as Fe^2+^, exists in a deeper layer of mussel thread, while Fe in higher oxidation state, such as Fe^3+^, tends to be enriched on the thread surface. C) Fe^2+^ and Fe^3+^ distributions as a function of the depth from the cuticle surface.

A recent finding reported that byssus threads were formed through two separate steps.^5^ First, the cuticle was assembled in an acidic environment with pH ∼ 3, and in the next step metals were infiltrated into the already assembled cuticle, leading to the mfp‐1 protein polymerization and iron‐DOPA crosslinking. Metal ions would interact with DOPA in both Fe^2+^ and Fe^3+^ forms, but there were more Fe^2+^‐DOPAs in the inner cuticle because of the acidic environment.^15^ Since the outer cuticle would form Fe^3+^, while the inner cuticle would still keep its Fe^2+^ coordination, a clear gradient distribution from Fe^3+^ to Fe^2+^ along the cuticle depth was observed in the synchrotron radiation test, as shown in Figure 2A,B. Since this oxidization is a slow process, it can also explain why an older mussel thread has more Fe^3+^ in the cuticle compared with fresh one.

To understand the degradation and self‐healing mechanism of the mussel threads, tensile tests of single threads were carried out in oxygen, air, and nitrogen environments. Water vapor was also added in the environments to keep 100% relative humidity (RH) such that the mussel threads were tested in wet state with different oxygen contents in all the cases. Figure S7 in the Supporting Information shows the collection of all the stress–strain curves measured under different environments. Like other biological materials, curves also have large variations in strength and extensibility, but the average stress–strain curves (**Figure** **3**A) clearly show the difference. As demonstrated in Figure 3A, the thread had the highest strain up to 115% in nitrogen environment, but only achieved 91% and 70% in the air and pure oxygen, respectively. Figure 3B shows variations in their mechanical properties. In addition, the strength of the cuticle is also environmentally dependent, with similar trend as the extensibility—the strength reduces with increasing the oxygen contents—suggesting that the threads were degraded by reacting with oxygen. Our tensile test results also show that the modulus of proximal thread does not strongly depend on the strain rate (Figure S8, Supporting Information), which agrees with the previous experiment.^16^ We further tested the stress–strain behavior of the threads and their collagen core. As shown in Figure S9 in the Supporting Information, the stress of the intact thread can be up to 229 MPa while the collagen core alone only shows a maximum stress of 19.2 MPa.

**Figure 3:**
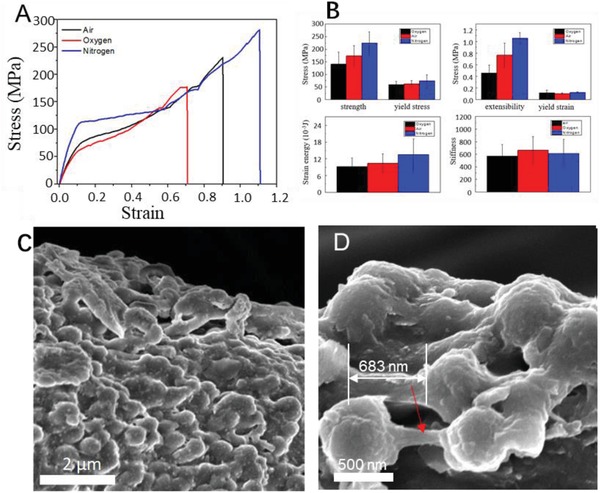
Force–strain curves and fractured surface of byssus threads, measured in different environments. A) Stress–strain curve of the thread under oxygen, air, and nitrogen environment, here the humidity was kept at 100% in all the tests. B) Comparison of the curves at different yielding stress, hardness, extensibility. C) SEM images of thread with tension strain of 90%, clearly crack can be observed on matrix. D) High‐resolution SEM images give the evidence of matrix bridging the gap between the granules during the tension process.

The fracture surfaces of the tested cuticles were examined with SEM. Figure 3C shows the SEM image of the cuticles at a strain of 30%. Cracks were observed clearly at the cuticle surface between granule and matrix, where the granules were torn from the matrix. When the strain increased to 50%, the cracks began to propagate along the tearing parts between matrix and granule. Matrix bonding, or bridging between granules was observed clearly, as shown in Figure 3D. Since bridging between the granule and matrix exists during the loading, the interfacial bonding must be strong. The matrix bridging during the tension process is a typical toughening mechanism in composite materials.

To understand the matrix–granule bridging behavior of the cuticle, we measured the mechanical properties of the transition area between the granule and matrix using AFM. AFM has been proved as a reliable tool for imaging as well as measurements of the various physical properties of composite bio‐structures in the air and aqueous environments.^17^ **Figure** **4**A shows an AFM image of cross‐section of the cuticle which includes sub‐microscale granules in polymer matrix. Using the AFM, we have measured for the first time the Young's modulus and hardness of single granule and matrix, as well as their interface. In the measurements, AFM probe with the size around 10 nm (Figure S10, Supporting Information) was used to made nanoindentation on granule and matrix under sea water (Figure 4B). For the interface measurement, the cross‐sections of fresh threads were tested under sea water by moving the probe across the interfaces between granules and matrix. Hardness and Young's modulus were extracted using modified hertz model (Equations (S1) and (S2) in Note S1, Supporting Information) that has been widely used to determine the Young's modulus of composites.^17^ As can be seen in Figure 4C,D, the hardness of the granules is 195 ± 5 MPa, which is 62.5% higher than that of the matrix (120 ± 5 MPa). The Young's modulus of the granules is 1.56 GPa, 32% higher than that of the matrix. These results are consistent with previous AFM measurements on the cuticle of *M. galloprovincials* (hardness of 133 ± 17.4 MPa and Young's modulus of 1.7 ± 0.1 GPa).^7^ The surfaces of the samples were further measured under SEM images and no obvious deformation was observed (Figure S11, Supporting Information). The interface is strong and no separation between the matrix and granule was observed during the indentation. This strong interface, together with matrix bridging, may contribute to the high strength and toughness of the mussel cuticle.

**Figure 4:**
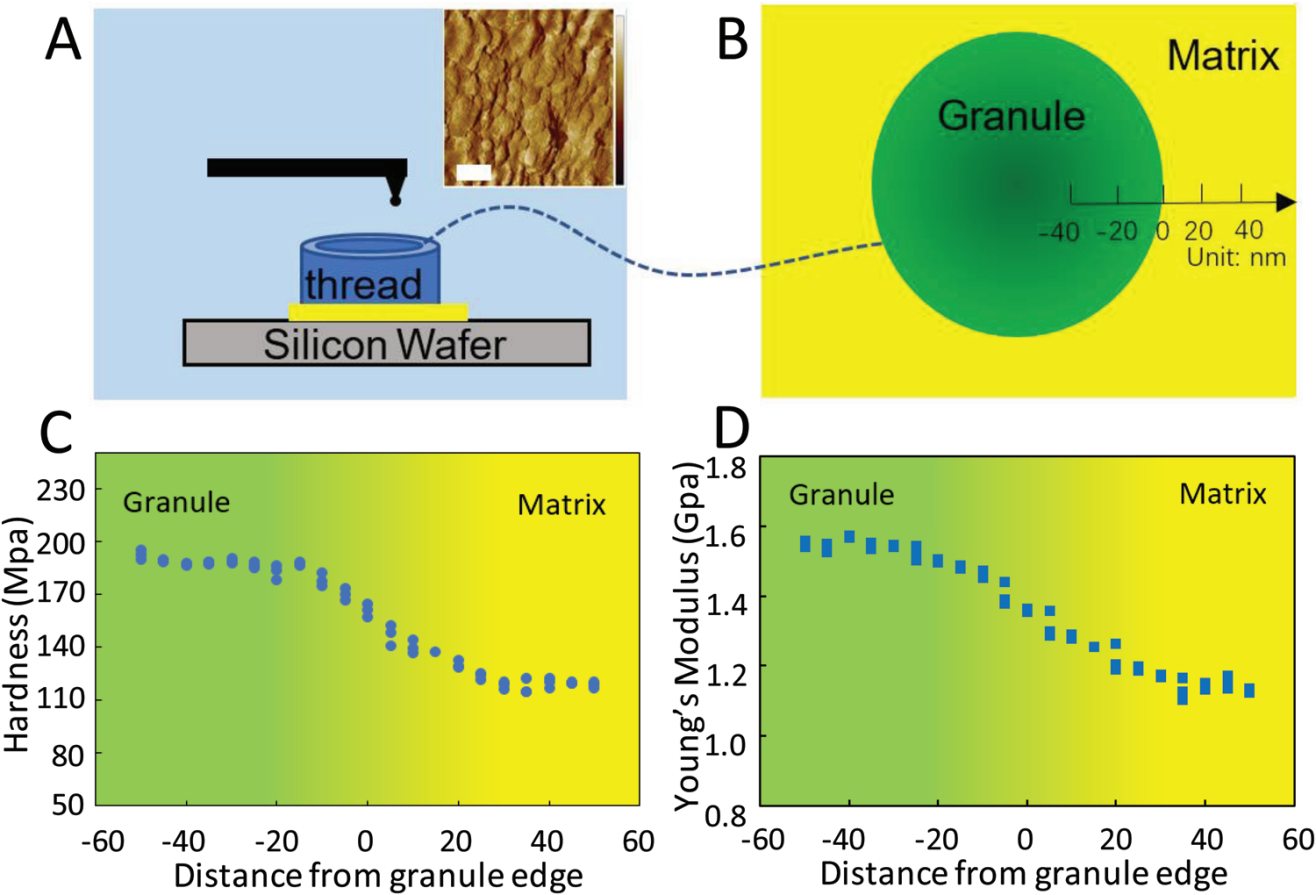
AFM image and measurement of hardness, and Young's modulus of cuticle across the granule–matrix interface. A,B) Setup of the AFM indentation test on granule and matrix, inset upright: AFM image of cut the cross‐section of a fresh thread, scale bar: 500 nm and C) hardness, and D) Young's modulus across the interfaces from granule to matrix, measured by AFM indentation. Both the hardness and Young's modulus decrease gradually across the interface from the granules to the matrix.

To fundamentally understand the mechanism of mechanical response of mussel threads to different environments, multiscale modeling was developed at the macro and molecular scales. First, the finite element modeling of whole byssal threads (Note S2, Supporting Information) was carried to determine the mechanical properties of the collagen core and cuticle coating (Figure S12, Supporting Information). By fitting the mechanical behaviors of intact and cut byssal threads (Figure S12c, Supporting Information), it was found that Ogden hyperelastic model, which is widely used for soft biological materials,^18^ well described the mechanical behavior of the core. While the collagen core bares the most of the loading in the tensile test, the cuticle plays an important role in determining the strength of the whole threads. Our simulations show that a through‐thickness crack in the cuticle could reduce the thread extensibility (strength) by up to 20% due to the stress concentration induced by the cracks in the hard cuticle. On the other hand, the intact cuticle can protect the core by arresting preexisting cracks in it. Therefore, it is likely that the mechanical properties of the threads reduced because the cuticle degrades in the oxygen‐containing environments. Moreover, oxygen may diffuse through the damaged area of the cuticle and interact with the collagen core to further damage the cuticle. In contrast, in the inert environment (e.g., N_2_), the oxygen‐damaged cuticle is healed by removing the adsorbed oxygen, and the core is well protected from the crack attack in the cuticle.

To understand how the cuticle and collagen core degrade at the molecular scale in the oxygen‐containing environments, we further performed the first‐principles calculations, using the density function theory (DFT) methods, and determined the electronic structures and chemical reaction pathways under different environments (Note S3, Supporting Information). Although mytilus byssus contains roughly 25–30 different proteins, only one protein, mfp‐1, is associated with certainty with byssal cuticles.^12^ We therefore exclude the role of the internal redox modulators such as thiols in the oxidation/reduction reactions. The reaction cycle of the redox from “damage” to recovery under various environments is illustrated in **Figures** **5**A, and bond forming and breaking in the cuticle during these processes are schematically shown in Figure 5B. First, we analyzed the reactions between oxygen and mussel cuticle when it is exposed to oxygen‐containing environments. In such environments, oxygen may absorb and react with the iron in the cuticle to weaken and even break the bonds between the iron and DOPA. As illustrated in Figure 5A‐b, O_2_ could adsorb on (bis‐)Fe‐DOPA coordination complex (Fe^2+^DOPA_2_) weakening the Fe‐DOPA bonds. The change in Gibbs free energy for the oxygen adsorption was calculated, yielding 0.056 and −0.06 eV in a neutral and a weak acidic media, respectively (Table S1, Supporting Information). The nearly zero free energy change indicates that the reaction is thermodynamically possible. The O_2_ absorption changes the electronic/bonding structures of Fe^2+^‐DOPA_2_ (Figure 5C and Figure S10, Supporting Information). The measurements of the Fe‐DOPA bonds (Table S2, Supporting Information) show that the bond length is increased due to the adsorption of O_2_ on Fe^2+^, which would weaken the Fe‐DOPA crosslinks, consequently reducing the mechanical properties of mussel threads. In addition to the adsorption of oxygen on Fe ions, we also calculated the possible adsorption of oxygen on DOPA chain (e.g., carbon, oxygen), but our results showed that oxygen molecule could not adsorb on the surface of the DOPA. Therefore, DOPA chains will remain intact in the oxygen‐containing environments.

**Figure 5:**
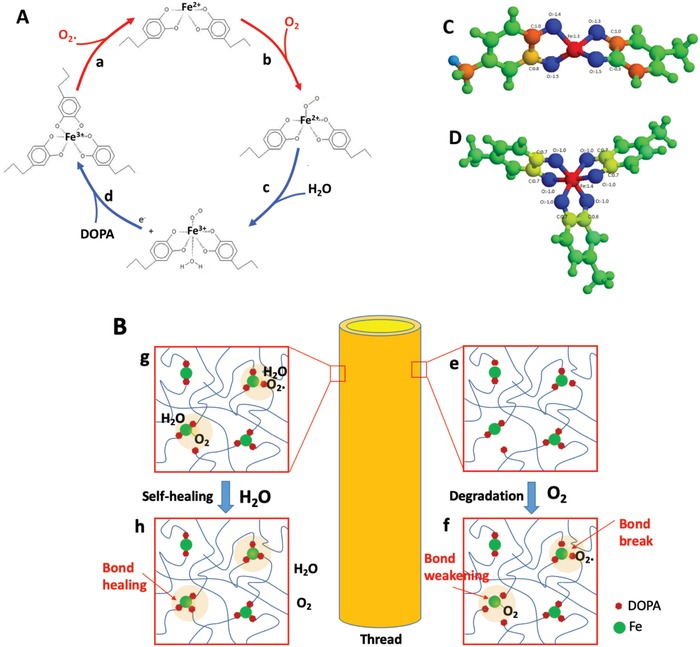
Molecular reaction mechanisms and coordination change in the degradation and self‐healing processes of cuticle. A) Proposed molecular mechanism of mussels, including the whole cycle of degradation and self‐healing. In the degradation process in the air, a) Fe^3+^DOPA_3_ reacts with a radical coupling O_2_ to form Fe^2+^DOPA_2,_ catalyzed by catechol dioxygenase enzymes, and b) O_2_ adsorbs on Fe^2+^DOPA_2_, weakening the Fe‐DOPA bonds. In the self‐healing process, c) one water molecule absorbs on the iron in O_2_ adsorbed (O_2_‐Fe^2+^)DOPA_2_ complex to form (H_2_O‐Fe^2+^‐O_2_)DOPA_2_ with an electron transfer, and d) a DOPA reacts with (H_2_O‐Fe^3+^‐O_2_)DOPA_2_ to form Fe^3+^DOPA_3_ by replacing the adsorbed O_2_ and H_2_O on the iron. B) Proposed microstructural changes in the degradation and self‐healing processes: e,f) O_2_ adsorption on iron, leading to bond weakening and break and g,h) H_2_O adsorption on iron, resulting in bond restoring and healing. Bader effective charge distribution of C) Fe^2+^DOPA_2_ and D) Fe^3+^DOPA_3_. The green, yellow, blue, and red colors refer to negative, positive, highly negative, and highly positive charges, respectively. The numbers are the values of the charges.

In oxygen‐containing environments, it is possible that (tris‐)Fe‐DOPA coordination complex (Fe^3+^DOPA_3_) is transformed into Fe^2+^DOPA_2_ through oxygen reduction reaction. In this transformation (Reaction (a) in Figure 5A), a radical oxygen coupling O_2_ would adsorb on a DOPA chain and break the bond with the iron through valence tautomerism, catalyzed by catechol dioxygenase enzymes that exist in the threads.^19^ After the reaction, the Fe^3+^DOPA_3_ transfers into Fe^2+^DOPA_2_. As an iron‐DOPA bond is broken, the 3D Fe^3+^DOPA_3_ crosslinking structures now become 2D Fe^2+^DOPA_2_ linear structures on the surface of the cuticle. However, the degradation process can only occur when a radical oxygen coupling O_2_ is generated by, e.g., light radiation.

In the aqueous environments, the damaged area of cuticle could be healed through oxygen evolution reaction (Reactions (c) and (d) in Figure 5A), the oxygen molecule would desorb from the iron ions in the iron‐DOPA complex to form Fe^2+^DOPA_2_ since the change in Gibbs free energy of the oxygen dissociation is −0.14 eV in sea water (pH = 8.1) (Table S2, Supporting Information). Furthermore, a DOPA would react with Fe^2+^DOPA_2_ to form Fe^3+^DOPA_3_, with a change in Gibbs free energy of −0.47 eV in sea water, suggesting that the reaction could occur spontaneously. After this reaction, the damaged polymer chains are healed. The mechanical properties of the mussel threads are therefore recovered due to the healing. It should be noted that the first reaction is the key step in the healing process since sea water not only reduces the reaction energy but also promotes the electron transfer between the iron ions and the ions in the sea water. Therefore, sea water effectively protects the mussel threads from the oxygen attack, or promotes the healing of damage if any.

The first‐principles calculations can also explain our experimental results that Fe^3+^ were enriched on the thread surface (Figure 2). After the formation of byssus threads, Fe^2+^‐enriched thread tissue reacts with sea water to further polymerize into Fe^3+^‐enriched cuticle, making the outer layer even harder. According to the DFT calculations, these reactions occur spontaneously in the presence of water. After the dense hard shell is formed due to the reaction, it reduces ion penetration into the inner part of cuticle, thus forming structures with more Fe^2+^ inside the cuticle. The gradient distribution of Fe^2+^ across the thickness of the cuticle leads to functionally graded mechanical properties due to different mechanical properties of the Fe^3+^DOPA_3_ and Fe^2+^DOPA_2_ complexes. As mentioned before, this functionally graded structure in the mussel threads may play an important role in enhancing the strength and extensibility, which have been observed in various biological structures such as bamboos and teeth.^1^

In summary, we have found, for the first time, that different coordinated Fe^3+^ and Fe^2+^ ions distribute gradually across the thickness of the cuticle in mussel threads, which leads to a functionally graded cuticle. The mechanical behaviors of the mussel thread were studied under inert nitrogen, air, and pure oxygen environments, and the results show that both strength and extensibility of the threads decrease with increasing oxygen contents. The degradation of the mechanical properties can be recovered by putting it into sea water. The first‐principles and finite element calculations were performed to understand the degradation and self‐healing mechanisms of the mussel threads at the molecular level. The DFT results show that the oxidation of Fe^2+^DOPA complexes can occur spontaneously in the oxygen‐containing environments, leading to degradation of the threads. Self‐healing takes place when spraying seawater on the threads because of the restoration of Fe^3+^‐DOPA complexes from the O‐adsorbed Fe^2+^DOPA complexes with water interaction. Our DFT calculations also explain why there is gradient distribution of Fe^2+^ in the cuticle, which may contribute to the overall properties of the threads. These findings provide insight into the strengthening and self‐healing mechanisms of the mussel cuticle and may have implication in designing/fabricating bioinspired materials with high strength, high toughness, and self‐healing capacity.

## Experimental Section

*Sample Preparation*: Permission for animal experiments was obtained from the General Hospital of Chinese People's Liberation Army. Mussel byssal threads from *Mytilus californianus* were harvested from mussels grown in a tank of flowing sea water from the Santa Barbara channel. Threads were washed, and the distal portions of threads were dissected from the rest of the thread and stored in distilled water prior to further experimentation.

*Tensile Testing of the Mussel Threads and Cuticle*: Tensile tests of mussel thread were carried using Bionix 200 tensile tester by MTS (MTS, Eden Prarie, USA) at 100% humidity environment condition, and the speed of all the tension tests was carried at 10 mm s^−1^ unless noted. While 100% humidity environment condition was kept, oxygen, air, and nitrogen were filled into the testing chamber before those tension tests. All the thread diameters and sizes were exampled before and after the tests. The hardness and Young's moduli values for the granule and matrix inside the cuticle were obtained by AFM nanoindentation test. The spring constant of the probe 5.4 N m^−1^ with a diameter of 2 nm was used (high density carbon tip, Bruker., Inc.). The indentation tests were carried out on cross‐sections of fresh threads with test specimens submerged in sea water.

*TEM Imaging*: Threads were fixed in glutaraldehyde for 2 h (*T* = 4 °C), PBS (phosphate‐buffered saline) buffer rinsed two times, and then fixed in osmic acid for 2 h, followed by dehydration in acetone under standard protocols, then soaked in 1:1 pure acetone and Epon 812 epoxy resin mixed for 30 min for 2 h. Then sample was embedded in Spurrs resin (Ted Pella, Redding, California) and microtome to produce thin sections of 100 nm, following standard protocols. Micrographs were obtained using a JEOL 123 TEM (Beijing, China) operated at 80 kV.

*XANES Examination*: XANES and near edge X‐ray absorption fine structure ( NEXAFS) spectra experiments were performed at the photoemission end‐station at beamline BL10B in the National Synchrotron Radiation Laboratory (NSRL) in Hefei, China. This beamline was connected to a bending magnet and equipped with three gratings that cover photon energies from 100 to 1000 eV with a typical photon flux of 1 × 10^10^ photons s^−1^ and a resolution (*E*/Δ*E*) better than 1000 at 244 eV. The end station was comprised of four chambers including analysis chamber, preparation chamber, high pressure reactor, and load‐lock chamber. The base pressures were 2 × 10^−10^, 2 × 10^−10^, 5 × 10^−9^, and 5 × 10^−9^ mbar, respectively. The analysis chamber was connected to the beamline and equipped with a VG Scienta R3000 electron energy analyzer, a twin anode X‐ray source (Mg Ka and Al Ka), an UV light source, a rear‐view optics for low energy electron diffraction, and a high precision manipulator with four‐degree‐of‐freedom (*X*, *Y*, *Z*, *R* for changing polar angle (θ)). The preparation chamber was comprised of an Ar^+^ sputter ion gun, a quartz crystal microbalance, a quadrupole mass spectrometer (Pfeiffer QMS220), a high precision manipulator with four‐degree‐of‐freedom (*X*, *Y*, *Z*, and *R*), and several evaporators. The high‐pressure reactor performed the in situ high pressure experiments under different atmosphere condition, which had a maximum range of 2 MPa and a highest temperature of 650 °C.

The Fe XANES spectra were measured at the photoemission end‐station at beamline BL10B in the National Synchrotron Radiation Laboratory (NSRL) in Hefei, China. A bending magnet was connected to the beamline, which was equipped with three gratings covering photon energies from 100 to 1000 eV. In this experiment, the samples were kept in the total electron yield mode under an ultrahigh vacuum at 5 × 10^−10^ mbar. The resolving power of the grating was typically *E*/D*E* = 1000, and the photon flux was 1 × 10^−10^ photons s^−1^. Spectra were collected at energies from 700 to 740 eV in 0.2 eV energy steps. The NEXAFS raw data were normalized by a procedure consisting of several steps. First, the photon energy was calibrated from the 4f spectral peak of a freshly sputtered gold wafer. Then a line was substrated to set the pre‐edge to be zero. Finally, the spectra were normalized to yield an edge‐jump to one.

In situ XPS measurements were performed at the photoemission end‐station at beamline BL10B in the National Synchrotron Radiation Laboratory (NSRL) in Hefei, China. Briefly, the beamline was connected to a bending magnet and covered the photon energies from 100 to 1000 eV with a resolving power (*E*/Δ*E*) better than 1000. The end‐station was composed of four chambers, i.e., analysis chamber, preparation chamber, quick sample load‐lock chamber, and high pressure reactor. The analysis chamber, with a base pressure of < 5 × 10^−10^ Torr, was connected to the beamline and equipped with a VG Scienta R3000 electron energy analyzer and a twin anode X‐ray source. The high‐pressure reactor housed a reaction cell where the samples could be treated with different gases up to 20 bar and simultaneously heated up to 650 °C. After the sample treatment, the reactor could be pumped down to high vacuum (<10^−8^ Torr) for sample transfer. In the current work, the sample was deposited on the system to analysis chamber for XPS measurement without exposing to air.

*DFT Simulations*: A series of models was developed to simulate the reactions of MFP‐Fe‐6O and MFP‐Fe‐4O with various oxygen ligands. DFT with Hubbard (DFT + U) was carried out using soft projector‐augmented wave pseudopotentials and the Perdew–Burke–Ernzenhof exchange correlation functional, as implemented in the VASP code. The plane wave kinetic energy had a high cut of energy of 550 eV throughout the computations and a vacuum spacing of at least 20 Å in the *z*‐direction. The k‐point setting of Brillioun zone was obtained by 3 × 3 × 3 grid generating meshes with their origin point at the gamma point. Moreover, all the spin‐polarized calculations were converged to 0.01 eV Å^−1^ for all surfaces and the geometries.

*Confocal Laser Scanning Microscopy (CLSM) Imaging*: Fresh mussel was cut and transferred to glycerine (Z99.5%, free of water, two times distilled, Carl Roth GmbH & Co. KG) and mounted using high‐precision cover slips. Then the autofluorescence of gecko setae was analyzed using the CLSM TCS SP8 (Leica, Germany) equipped with an inverted microscope and two solid‐state lasers (wavelengths: 405, 488, 633 nm) and Argon laser. For the visualization, a 40 × objective (HCX PL APO CS 40 × 1.30 OIL, 506358, Leica, Germany) was used.

## Conflict of Interest

The authors declare no conflict of interest.

## Supporting information

Supporting Information
