# Supplementary material for: Metal Coordination‐Mediated Functional Grading and Self‐Healing in Mussel Byssus Cuticle
Source: Adv Sci (Weinh). 2019 Oct 24;6(23):1902043. doi: 10.1002/advs.201902043 (PMC6891911; doi:10.1002/advs.201902043)
Supplement: Supplementary file 1 — Supporting Information [file ADVS-6-1902043-s001.pdf]

Copyright WILEY-VCH Verlag GmbH & Co. KGaA, 69469 Weinheim, Germany, 2019.

Supporting Information

for *Adv. Sci.,* DOI: 10.1002/advs.201902043

Metal Coordination-Mediated Functional Grading and Self-Healing in Mussel Byssus Cuticle

Quan Xu, Meng Xu, Chun-Yu Lin, Qiang Zhao, Rui Zhang, Xiaoxiao Dong, Yida Zhang, Shouceng Tian, Yu Tian,* and Zhenhai Xia**

Metal Coordination-Mediated Functionally Grading and Self-Healing in Mussel Byssus Cuticle

Quan Xu,[a=*] Meng Xu,[b=] Chun-Yu Lin,[c] Qiang Zhao,[d] Rui Zhang,[a] Xiaoxiao Dong,[a] Yida Zhang,[a] Shouceng Tian,[a] Yu Yian,[e*] Zhenhai Xia[c,f*]

[a] *State Key Laboratory of Heavy Oil Processing, China University of Petroleum-Beijing, Beijing,102249, China*
[b] *Department of Orthopedics, General Hospital of Chinese People's Liberation Army, Beijing 100853, China*
[c] *Department of Materials Science and Engineering, University of North Texas, Denton, Texas 76203, USA*
[d] *School of Chemistry and Chemical Engineering, Huazhong University of Science&Technology Wuhan 435000, China*
[e] *State Key Laboratory of Tribology, Tsinghua University, Beijing 100084, China*
[f] *School of Materials, Northwestern Polytechnical University, Xi'an 710072, China*

Correspondence and requests for materials should be addressed to:
Q. Xu (email: xuquan@cup.edu.cn); Y. Tian(Email: tianyu@mail.tsinghua.edu.cn)
Z. Xia (Email: Zhenhai.Xia@unt.edu)

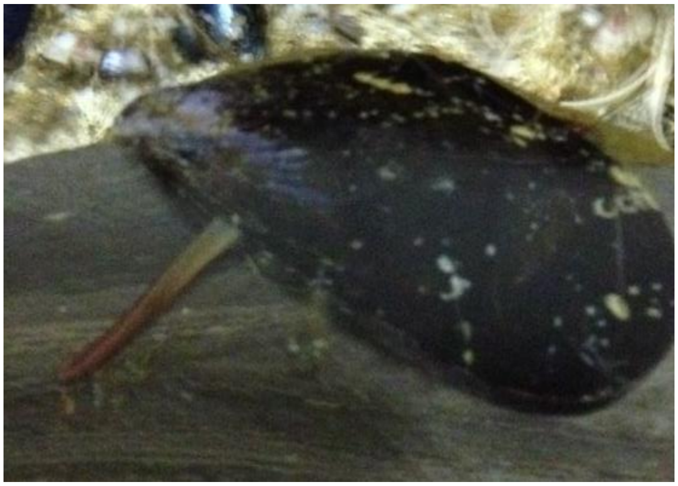

Figure S1. A photo image capturing a mussel forming thread.

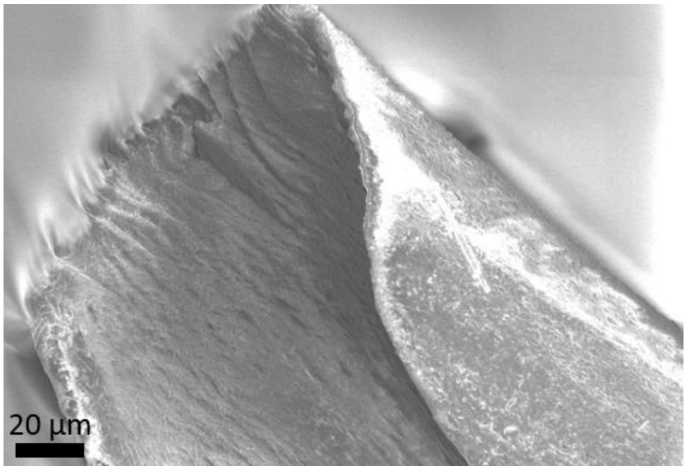

Figure S2. SEM image of the cuticle coating at the temperature of 10 K, where the inside fibril was shirked at that temperature. The cuticle shortens less than 10% along the length at the temperature decrease from room temperature to 10 K. As in, the inside fibers shirked while the cuticles kept its shape and formed a hollow tube.

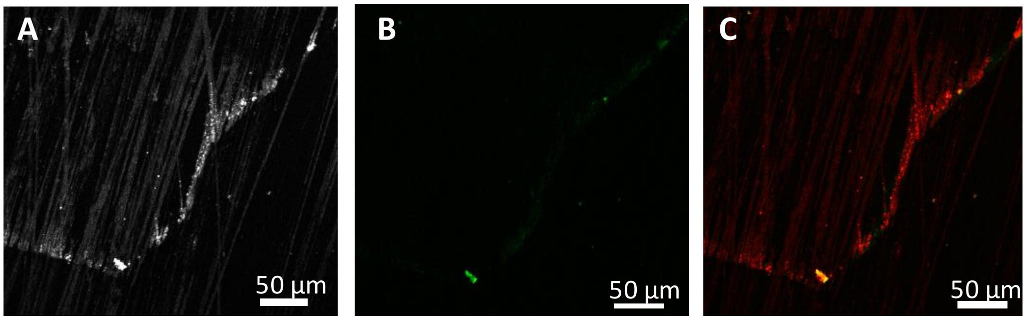

Figure S3. **Confocal laser microscope image of the thread.** Confocal laser microscope image of the cross section of the thread at (A) 405, (B) 488, and (C) 633 nm auto fluorescence wavelengths, respectively.

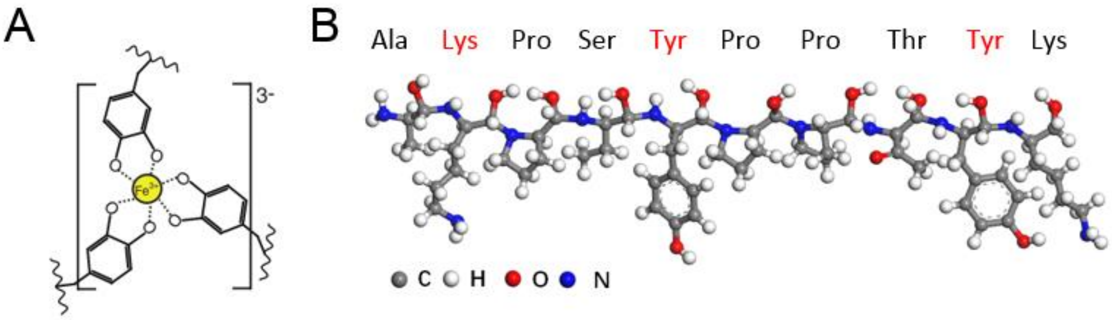

Figure S4. Illustration representatives of DOPA-iron coordination complex. (A) The hexadentate mononuclear tris DOPA-iron coordination complex proposed to cross-link mfp-1 in the byssus coating, and (B) Sequence of mussel foot protein.

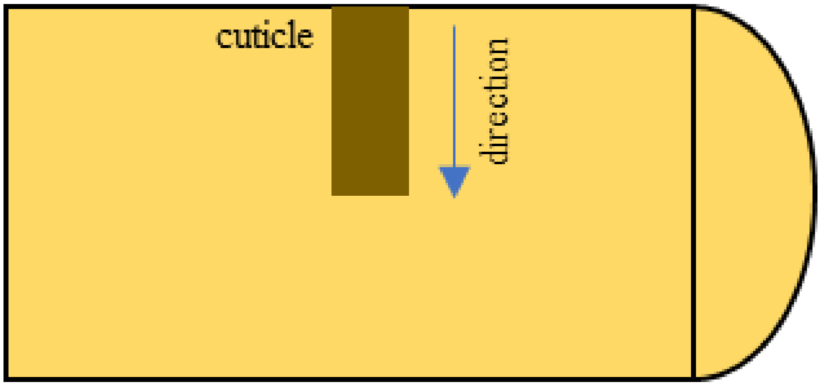

Figure S5. The schematic of the etching direction along the cuticle thickness.

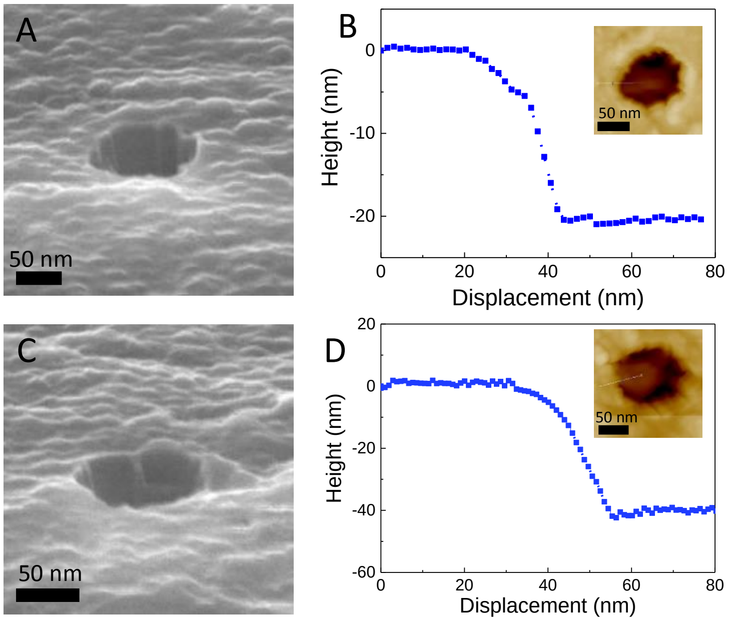

Figure S6. The SEM images of the etching thread with depth at (A) 20 nm and (C) 40 nm. The AFM images (insets) and height profiles of the etching thread with depth at (B) 20 nm and (D) 40 nm.

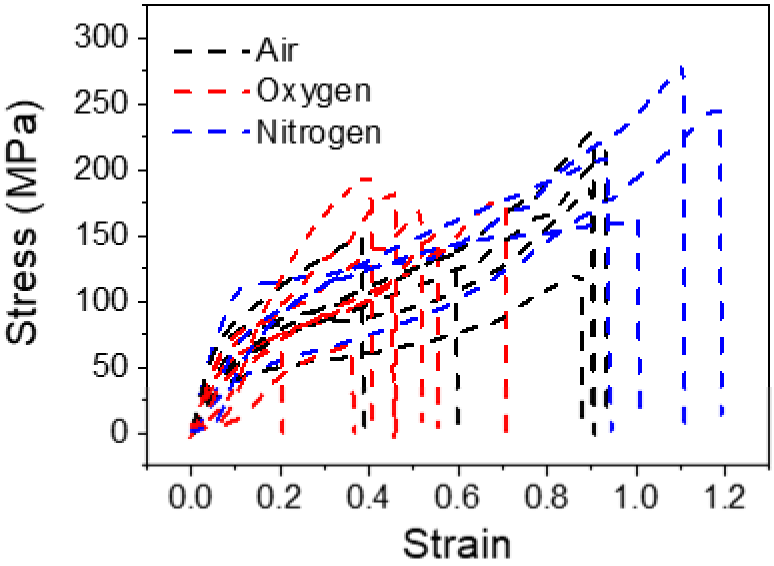

Figure S7 Stress-strain curve of the thread under oxygen, air and nitrogen environment, here the humidity was kept at 100% in all the tests.

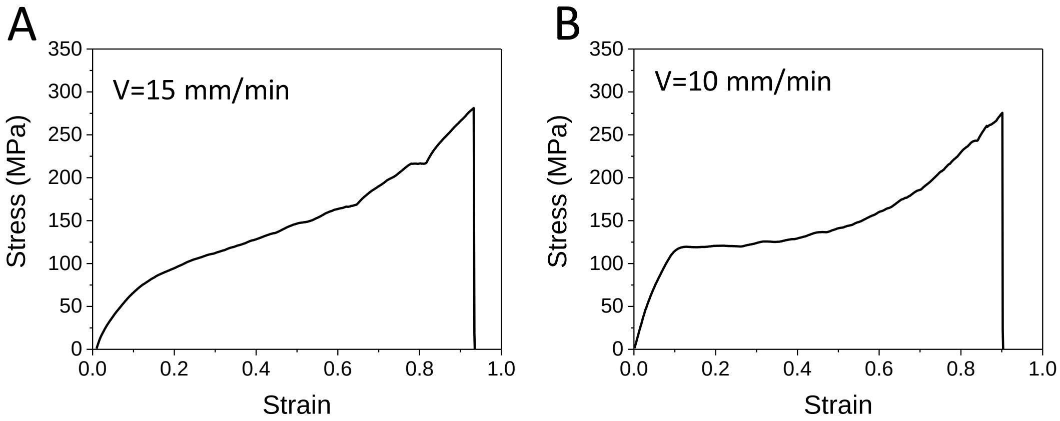

Figure S8. Tensile test of cuticle under different strain rate. Stress-strain curves of cuticle under (A) 15mm/min and (B) 10 mm/min tension velocity.

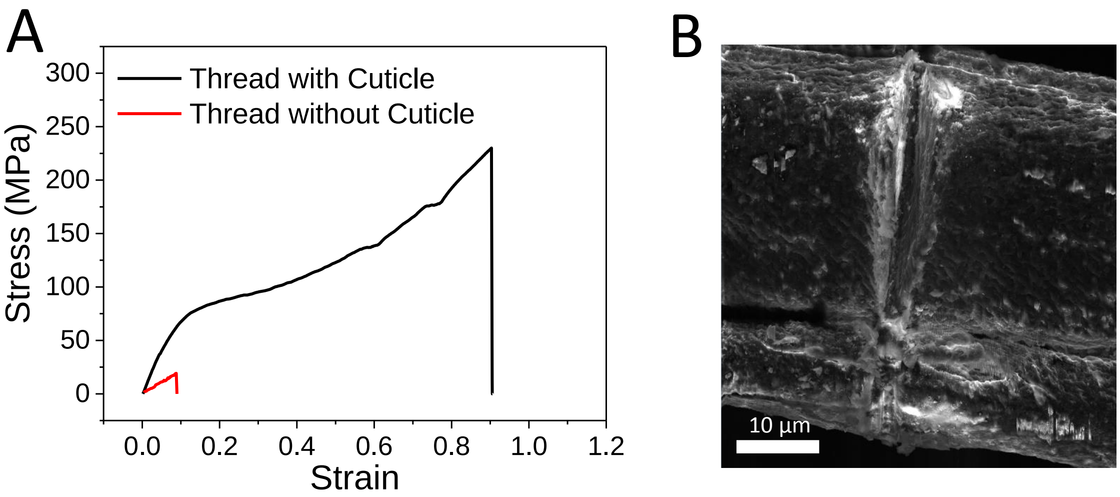

Figure S9 (A) Stress-Strain curves of the thread and that with a ring cutting, and (B) the SEM image of the thread with a ring cutting

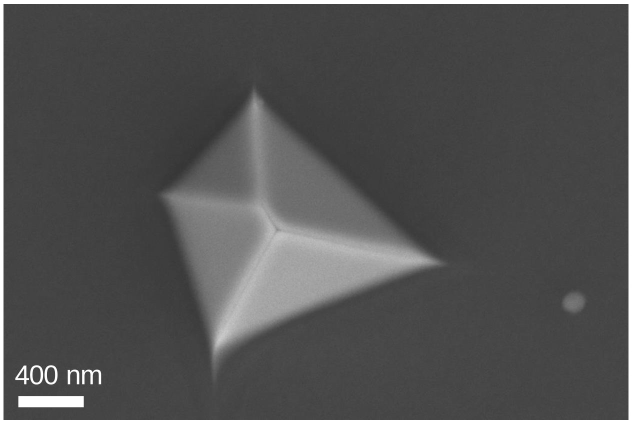

Figure S10. SEM images of AFM probe.

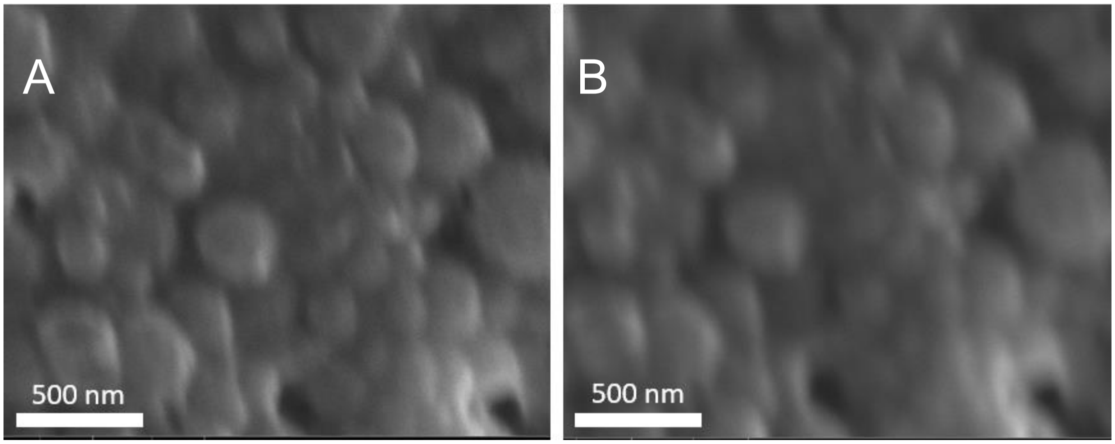

Figure S11. SEM images (A) before and (B) after the indentation test.

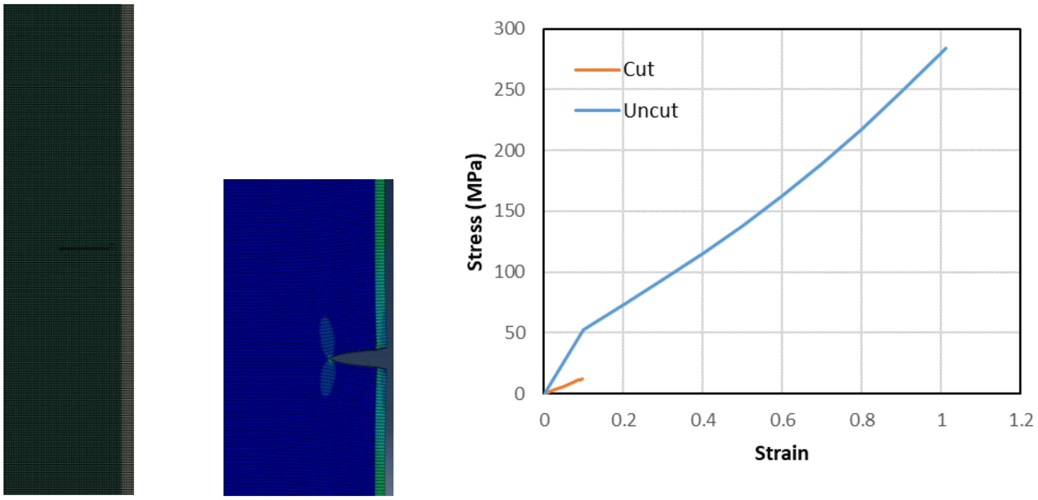

A B C

Figure S12 (A) Finite element models of mussel bypass thread with 1.6 μm thickness cuticle, (B) finite element model of the thread with a crack, and (C) stress-strain curves of the undamaged and damaged threads.

Note S1: Calculation of Hardness and Young's modulus

The Hertz model was used to analysis the Hardness and Young's Modulus of the mussel granule and matrix. Equation for the Hardness measurement is written[S1]

$$\mathrm{H} = \frac{F}{2\pi R_c \delta} \qquad (S1)$$

where F being the force obtained from the FDCs, R_c the radius of the curvature of the cantilever tip (30 nm) and δ the indentation depth of the tip. For the Young's Modulus analysis:

$$\mathrm{F} = \frac{E}{1-\nu^2} * \frac{4\sqrt{R_c}}{3}\delta^{\frac{3}{2}} \qquad (S2)$$

where E the Young's Modulus, and v the assumed Poisson ratio set to 0.5,

Note S2 Modeling of mechanical behavior of mussel byssus threads

An axisymmetric FE model was created to simulate tensile characteristics of mussel byssus thread. Because of the tissue incompressibility, the hybrid elements (with pressure as an additional variable) were chosen in ABAQUS (v6.12, Simulia, Providence, RI). An Ogden hyperelastic constitutive definition was adopted for describing the mechanical behaviors of the core of the thread, while the cuticle was described by an elastic-plastic materials model. In the Ogden hyperelastic model, strain energy W is a function of deviatoric principal stretches (λ_n):[S7]

$$W(\lambda_1, \lambda_2, \lambda_3) = \sum_{i=1}^{N} \frac{2\mu_i}{\alpha_i^2}\left(\lambda_1^{\alpha_i} + \lambda_2^{\alpha_i} + \lambda_3^{\alpha_i} - 3\right) \qquad (S3)$$

where the coefficients μ_i and α_i predominately reflect the low-strain slope and the higher strain curvature, respectively, of the resultant stress–strain curve. In this work, we have chosen the first-order Ogden and relaxation model of N =1. By fitting the experimental results for a typical byssus thread, we had obtained materials parameters for thread core: Modulus (MPa) μ = 100 MPa, and α = 3.5. For the cuticle, Young's modulus E=2.5GPa, Poisson's ratio v = 0.45, and yield strength σ_y = 250 MPa.

The diameter of the threads in the model is chosen to be 50 μm, and the thickness of the cuticle is 1.6 μm. A cohesive model within the framework of XFEM was used to describe the crack propagation behavior. The failure displacement is 0.1μm, and the cohesive strength (principal stress) is in the range of 200-400 MPa.

Note S3: Calculation of the Gibbs free energy of the redox reactions

1.1 Reaction pathways

In oxygen-enriched environments, in the first step, a radical O_2 would adsorb on a DOPA chain and break the bond from the iron through valence tautomerism catalyzed by catechol dioxygenase enzymes that exist in the threads.[S2-S3]After one DOPA is broken from Fe-DOPA complex, the oxygen continues reacting with the complex. The reactions between oxygen and iron-DOPA complex are assumed to follow the following reaction path:

$$(Fe^{2+})R_2 + O_2 \rightarrow (Fe^{2+}O_2)R_2 \quad \text{(S3)}$$

$$(Fe^{3+})R_3 + O_2 \rightarrow (Fe^{3+}O_2)R_3 \quad \text{(S4)}$$

where R represents quinone molecule (DOPA).

In the self-healing process, the reaction can be separated into two steps. In the first step, water adsorbs on Fe-DOPA complex with an electron transfer.

$$(Fe^{2+}O_2)R_2 + H_2O \rightarrow (H_2OFe^{3+}O_2)R_2 + e^- \quad \text{(S4)}$$

where e^- is the one electron. In the second step, a $(Fe^{2+}O_2)R_2$ reacts with a DOPA to form tris iron-DOPA complex.

$$(H_2OFe^{3+}O_2)R_2 + R \rightarrow (Fe^{3+})R_3 + O_2 + H_2O \quad \text{(S5)}$$

The Gibbs free energy is calculated using the density functional theory (DFT). For a reaction A+B→C, the reaction energies are calculated with following equations.

$$\Delta E = E_C - E_A - E_B \quad \text{(S6)}$$

where E_A and E_B, and E_C are the reaction energy of reactants and products. The Gibbs energy of the reaction is determined with follow equation,[S4]

$$\Delta G = \Delta E + \Delta ZPE - T\Delta S + \Delta G_{pH} \qquad \text{(S7)}$$

where ΔZPE is the zero-point energy difference, T is the temperature, and ΔS is the entropy capacity, ΔG_{pH} is the value of correction of H+ free energy with a value of 0.828 in alkaline environment (pH=14) and zero in acidic media (pH=0).[S6]

$$\Delta G_{pH} = -k_b T ln[H^+] \quad = k_b T ln[10] pH \qquad \text{(S8)}$$

The energies of these terms for different species are listed in Table S3.

2. Supplementary Tables

Table S1. Possible Reactions in degrading and healing processes and the Gibbs free energy of the reactions

Process	Reactions	Environments	ΔG(eV)
**Degradation**	$(Fe^{3+})R_3 + {}^{\cdot}O_2^- \rightarrow (Fe^{2+})R_2 + RO_2$	UV/enzymes	-
	$(Fe^{2+})R_2 + O_2 \rightarrow (Fe^{2+}O_2)R_2$	Weak acidic(pH=5)	-0.06
		Pure water (pH=7)	0.056
		Sea water (pH=8.1)	0.12
**Self-healing**	$(Fe^{2+}O_2)R_2 \rightarrow Fe^{2+}R_2 + O_2$	Pure water (pH=7)	-0.056
		Sea water(pH=8.1)	-0.12
	$(Fe^{2+})R_2 + RO_2 + H_2O \rightarrow (Fe^{3+})R_3 +$	Pure water(pH=7)	-0.534
	$2OH^-$	Sea water(pH=8.1)	-0.47
	$(Fe^{2+})R_2 + R \rightarrow (Fe^{3+})R_3 + e^-$	Pure water(pH=7)	-0.534
		Sea water(pH=8.1)	-0.82

Table S2. Average bond length between Fe and DOPA

	$(Fe^{2+})R_2$	$(Fe^{2+}O_2)R_2$	$(Fe^{3+}O_2)R_3$	$(Fe^{3+})R_3$
**Average bond length (Å)**	2.00	2.04	2.06	1.93

Table S3. Gibbs free energy of each elements, the zero point energy (ZPE) corrections and entropy (TS)*

	E_0(eV)	ZPE(eV)	TS (eV)	ΔG(eV)
$(Fe^{2+})R_2$	-203.14	0.40	-0.14	-202.59
$(Fe^{2+}O_2)R_2$	-213.16	0.15	-0.13	-212.88
$(H_2OFe^{3+}O_2)R_2$	-227.79	0.81	-0.25	-226.73
**R**	-97.04	0.21	-0.08	-96.74
$(Fe^{3+})R_3$	-300.71	0.54	-0.01	-300.16
e^-				-0.47
O_2(g)				-9.93[S5]
H_2(g)	-6.76	0.27	0.41	-6.90[S5]
H_2O(0.035bar)	-14.22	0.56	0.67	-14.33[S5]

*The gas phase values are cited from Ref. 5, while the values for the adsorbed were taken from DFT calculations. Gas phase H_2O at 0.035 bar was used as the reference state because at this pressure gas phase H_2O is in equilibrium with liquid water at 300 K. The same values for the adsorbed species for all the models were used, as vibrational frequencies have been found to depend much less on the surface than the bond strength.

References

[S1] Iyer S, Gaikwad RM, Subba Rao V, Woodworth CD, Sokolov I. *Nat Nano* **2009**, 4, 389-393,

[S2] T.D. H. Bugg, and G.Lin *Chem Commun*, **2001,** 11**,** 941-952

[S3] J.J. Wilker *Cur Opin Chem Biol* 14, 276-83 **2010**.

[S4] M. Li, L. Zhang, Q. Xu, J. Niu, Z. Xia, *J Catal*, **2014**, 314, 66

[S5] P.W. Atkins, *Phys. Chem* **1998**, 485, 925–927 and 942

[S6] E. Skulason, G. S. Karlberg, J. Rossmeisl, T. Bligaard, J. Greeley, H. Jonsson and J. K. Nørskov, *Phys Chem Chem Phys*, **2007,** 9, 5158–5161

[S7] Z.J.Ma, S.H.Hu, J.S. Tan, N. Njus, Z.H. Xia, *J. biomedic. mater. Res. Part A*, **2013**, 9, 2718-2725
